# Development of a Nanoparticle-based Lateral Flow Strip Biosensor for Visual Detection of Whole Nervous Necrosis Virus Particles

**DOI:** 10.1038/s41598-020-63553-z

**Published:** 2020-04-16

**Authors:** Dimitra K. Toubanaki, Maritsa Margaroni, Athanasios Prapas, Evdokia Karagouni

**Affiliations:** 1 Immunology of Infectious Diseases Laboratory, Department of Microbiology, Hellenic Pasteur Institute, 127 Vas. Sofias Ave., 11521 Athens, Greece; 2 Department of Pathology of Aquatic Organisms, Veterinary Center of Athens, 25 Neapoleos Str, 15341 Agia Paraskevi, Greece

**Keywords:** Biosensors, Infectious-disease diagnostics

## Abstract

Effective analysis of pathogens causing human and veterinary diseases demands rapid, specific and sensitive detection methods which can be applied in research laboratory setups and in field for routine diagnosis. Paper lateral flow biosensors (LFBs) have been established as attractive tools for such analytical applications. In the present study a prototype LFB was designed for whole particles (virions) detection of nodavirus or fish nervous necrosis virus. Nodavirus is an important threat in the aquaculture industry, causing severe economic losses and environmental problems. The LFB was based on polyclonal antibodies conjugated on gold nanoparticles for signal visualization. Brain and retinas from fish samples were homogenized, centrifuged and the supernatant was directly applied on the LFB. Formation of a red test line was indicative of nodavirus virions presence. Nodavirus visual detection was completed in short time (30 min). Key factors of the LFB development influencing the assays’ detection limit were characterized and the optimum parameters were determined, enabling increased efficiency, excluding non-specific interactions. Therefore, the proposed LFB assay consists a robust, simple, low cost and accurate method for detection of nodavirus virions in fish samples. The proposed biosensor is ideal for development of a commercial kit to be used on aquaculture facilities by fish farmers. It is anticipated that disease monitoring and environmental safety will benefit from the simplification of time consuming and costly procedures.

## Introduction

Aquaculture is essential to cover fish-product demands, providing seafood in high quantities, and covering more than the half amount of fish consumed worldwide. This fact drives a strong demand for high production efficiency in aquaculture industry in order to cover the feeding needs of the world’s growing population, in the middle of an increasing environmental crisis^1,2^. As a result, the aquaculture industry has continuously increased profits in a high rate. However, outbreaks of diseases caused by infectious agents are significantly restricting intensified aquaculture. According to literature^3^, 22.6% of all disease outbreaks are caused by viruses. Among these, viral nervous necrosis (VNN), also named vacuolating encephalopathy and retinopathy or encephalomyelitis, is a devastating disease, which induces cell necrosis accompanied by vacuolation in fish retina and brain. Its clinical symptoms include changes in skin color with abnormal swimming, low feed ingestion and altered buoyancy in affected fish. The disease is caused by nervous necrosis virus (NNV) or nodavirus, affecting more than 30 different fish species, worldwide. VNN causes high mortalities (80–100% in several species e.g. European sea bass), emerging as a major problem especially in the Mediterranean area, since it cannot be prevented by vaccination or effective treatment^4–6^.

Fish nervous necrosis virus (belonging to *Betanodavirus* genus and *Nodaviridae* family) is icosahedral, and non-enveloped (∼25 nm in diameter). Its genome consists of two positive-sense single-stranded RNA molecules: RNA 1 (3.1 kb), which directs the synthesis of RNA-dependent RNA polymerase (100 kDa), and RNA 2 (1.4 kb), encoding the viral coat protein (42 kDa). The RNA1 segment also contains an RNA3 transcript which encodes the B1 and B2 non-structural proteins. Assessment of nodavirus genome has resulted in the following genotype classification: striped jack NNV, red-spotted grouper NNV, tiger puffer NNV and barfin flounder NNV^4,5^.

Several detection methodologies have been proposed for nodavirus, including virus isolation in cell cultures^7^, light- and electron-microscopy^4^, enzyme‐linked immunosorbent assay (ELISA), immunofluorescence antibody test, and molecular assays, i.e. *in situ* hybridization, polymerase chain reaction (PCR), reverse transcription PCR (RT‐PCR), and real time RT-PCR. Recently developed methodologies include loop-mediated-isothermal-amplification (LAMP) in conventional and real-time assays, or combined with microfluidic devices and laser-induced fluorescence detection technology. Other approaches rely on immunomagnetic reduction, magnetic beads conjugated to sequence-specific captured probes or molecular beacons, and gold nanoparticles based nucleic acid lateral flow biosensors, summarized in^8,9^.

Despite their advantages, the mentioned methods have drawbacks that restrict their use on site. In particular, PCR is based on expensive reagents and equipment, complicate sample preparation by well-trained analysts and is time consuming. On the other hand, ELISA-based methods use expensive kits, including labelled antibodies which are combined with cumbersome equipment^10,11^. All these demands limit their wide use for routine testing, especially in poor-equipped laboratories, as well as their accessibility and usability in field diagnosis. Therefore, development of a sensitive, easy-to-use, rapid, selective and robust, method, would be the only viable solution for routine nodavirus screening, ideally with application in the field.

Paper-based immuno-chromatographic or lateral flow immuno-assays or lateral flow biosensors (LFB) have proved to be very convenient tools with excellent accuracy, sensitivity and short assay times. They are based on solid-phase membrane with dried components which can be activated when a fluid sample is applied. LFBs combine the principles of capillary phenomenon and immune recognition reaction, they are intended for single use when an on/off signal is sufficient and they are disposable. Several nanoparticulate labels and detection platforms, including surface-enhanced Raman scattering, paper-based electrochemical components or hybrid paper/polymer microfluidic ELISAs, have been combined successfully with lateral flow and paper-based microfluidic platforms^12–15^. Antibody/oligonucleotide conjugated nanoparticles, such as colloidal gold, quantum dots, super-paramagnetic or silica nanoparticles, and other nanostructured materials, have been utilized as colored recognition elements. Among those nanoparticles, colloidal gold has been widely used because of its excellent optical and chemical stability^16,17^.

Lateral flow biosensors have been introduced for detection of proteins, mRNA, DNA, miRNA, heavy metals, chemical contaminants, pesticides and biological agents^17–21^. Detection of intact virus, i.e. whole virus particles (virions), is highly desirable for point-of-care testing platforms, such as LFBs^22,23^. Most approaches for nodavirus diagnosis are based on sample pretreatment for isolation and/or amplification of the target analyte, however the virion detection approach would enable virus detection without additional processing steps. Therefore, aim of this study was the development and validation of a sensitive lateral flow strip biosensor prototype for nodavirus detection in fish samples, based on rabbit polyclonal antibodies against nodavirus, conjugated with gold nanoparticles.

Most experimental investigations of lateral flow assays are focused on the end product, characterizing only a few test system parameters^24^. However, lateral flow assays sensitivity on virus detection has been estimated from 19% to 96%^25^. Thus, the low sensitivity in virus detection with lateral flow assays restricts their application as reliable assays for on-site analysis. In order to construct LFBs with higher sensitivity a reasonable procedure to follow would be based on the assay parameters improvement.

As mentioned above, aim of the present study was the development of nodavirus whole particle (virion) detection LFB, based on gold nanoparticles. Therefore, it is focused on the study of key factors on LFB development both in terms of gold nanoparticles (Au NPs) conjugates preparation and the lateral flow biosensor construction and performance: antibody – nanoparticle conjugation parameters were extensively studied, and the biosensor construction parameters, as well as the developing solution composition were optimized. The characteristics of the assay were assessed and the LFB was tested with real samples. In brief, brain tissue isolated from fish samples was homogenized and centrifuged. The supernatant was applied next to anti-nodavirus conjugated Au NPs and formation of a red test line was indicative of nodavirus particles presence. The visual detection was completed within 30 min.

## Materials and methods

### Reagents

SSN-1 cells were purchased from The European Collection of Animal Cell Cultures (Salisbury, UK). Fetal bovine serum (FBS) and Leibovitz L15-medium were from Biochrom (Berlin, Germany), and Whatman filters (PTFE, 0.22 μm) were from GE healthcare (Buckinghamshire, England). Penicillin, streptomycin and L-glutamine were from Gibco (Paisley, UK). Cell culture flasks Falcon Primaria were purchased from Becton Dickinson Labware (Franklin Lakes, USA) and flat bottom 96-well ELISA plates were obtained from Greiner (Kremsmunster, Austria). Micro bicinchoninic acid (BCA) protocol for protein quantification, Nab^TM^ Spin columns and Zeba^TM^ desalting columns were from Pierce (Thermo Fisher Scientific, Delaware, USA). Borax, dimethyl-sulfoxide, bovine serum albumin (BSA) and glutaraldehyde, were purchased from Applichem (Darmstadt, Germany). Eagle Minimum Essential Medium (EMEM), complete and incomplete Freund’s adjuvant (CFA and IFA, respectively), anti-rabbit IgG-horseradish peroxidase (HRP), poly-L-lysine, anti-rabbit IgG (whole molecule) antibodies, 3,3′,5,5′-tetramethylbenzidine (TMB) substrate, Au NPs stabilized in PBS (5, 10, 20, 30, 60 nm), or citrate buffer (40 nm), siliconized tubes and Tween-20 were obtained from Sigma-Aldrich (Steinhem, Germany). Monoclonal anti-nodavirus antibody was from Aquatic Diagnostics (Stirling, UK). Nitrocellulose membranes on laminated cards (HF240MC100 and HF180MC100), glass (GFCP000800) and cellulose fiber pads (CFSPOO1700) were all from Millipore (Billerica, MA, USA). Applichem or Sigma provided all common reagents.

Low salt washing buffer (LSWB-10×) contained, 200 mM Tris, 32.8 M NaCl, and 5 mL/L Tween-20, pH 7.4, and high salt washing buffer (HSWB-10×) consisted of 200 mM Tris, 5 M NaCl and 10 mL/L Tween-20, pH = 7.7. Developing solution consisted of 60 mL/L glycerol, 2 mL/L Tween-20 and 10 g/L sodium dodecyl sulfate (SDS) in phosphate-buffered saline (PBS: 0.14 M NaCl, 10 mM sodium phosphate, 2.7 mM KCl, and 1.7 mM potassium phosphate, pH 7.4). Deionized water using by Arium comfort system from Sartorius (Goettingen, Germany) was used for solutions preparation.

### Instrumentation

The thin layer chromatography (TLC) applicator Linomat 5 and Wincats software were from Camag (Muttenz, Switzerland). The desktop scanner HP scanjet G4050 was from HP (California, USA)^26^. The Nanodrop 2000 spectrophotometer was purchased from Thermo Fisher Scientific (Delaware, USA) and the ultraviolet–visible spectroscopy plate reader was the MRX model from Dynatech Laboratories (Chantilly, VA, USA).

### Fish samples

European sea bass (*Dicentrarchus (D.) labrax*) samples with or without VNN clinical signs were collected from sea-cage fish farms in Cyprus, Saronikos, Korinthian and Amvrakikos Gulfs. Dissected brain and retinas were isolated aseptically and stored in sterile polypropylene tubes at −80 °C until use^8^.

### Ethical statement

The collection of biological samples from fish farms was conducted by licensed personnel of the respective aquaculture facility. All qualified personnel were previously informed of the purpose of the study, the confidentiality of the data, and their voluntary participation. The present study was approved by the Hellenic Pasteur Institute Animal Bioethics Committee regulations according to Greek (PD 56/2013) and EU (Directive 63/2010) legislation for the protection, care and use of animals used for scientific purposes and all samples used were euthanized in the sites of fish farming according to the ethical principles and other requirements of the law^27^.

### Virus preparation and titration

Nodavirus was isolated from naturally infected *D. labrax* fish. All infected samples were verified by RT-PCR^9^. The virus was propagated in SSN-1 cells as follows: fish brains were homogenized either in EMEM or Leibovitz L15-medium containing 10% FBS. Centrifugation (4,000 × g, 15 min, 4 °C) of the homogenates (10% w/v) resulted in supernatant, which was filtered through 0.22μm filters and was inoculated on cell cultures. Following inoculation, the SSN-1 cells were cultured at 26 °C, in Falcon Primaria cell culture flasks containing Leibovitz’s L15-medium, supplemented with 100 μg/mL streptomycin, 100 u/mL penicillin, 2 mM glutamine and 10% FBS.

Virus titration was performed on monolayers of SSN-1 cells grown in a 96-well plate. Viral suspensions were prepared with 11-fold serial dilutions in EMEM supplemented with 10% FBS. Quadruplicates of 50 µL of each dilution were added in a 96-well plate seeded with SSN-1 cells. Cultures were incubated at 26 °C for 6 days. During this period, the cell monolayers were observed for the appearance of cytopathic effect and the final titer, expressed as TCID_50_/mL, was estimated by the end-point titration method^28^.

### Rabbit anti-nodavirus polyclonal antibodies preparation

Female New Zealand rabbits were used for anti-nodavirus polyclonal antibodies production with prior approval by the Animal Bioethics Committee of the Hellenic Pasteur Institute (Athens, Greece) according to the EC Directive 1986/609 and the National Law 1992/2015. Rabbits were reared in institutional facilities under specific pathogen-free conditions, receiving a diet of commercial food pellets and water *ad libitum*^29^. For immunization, the rabbits were inoculated with culture supernatant of nodavirus infected SSN-1 cells. In immunization scheme 1, one subcutaneous injection containing 300 μg of antigen emulsified with CFA was administered in final volume of 1000 μL. Three weeks later, a subcutaneous booster [300 μg of antigen emulsified with IFA] was injected. A final booster of 300 μg antigen without any adjuvant was given 21 days later. Blood was collected from rabbit marginal ear vein 15 days after the final booster, the serum was isolated by centrifugation at 800 × g for 20 min and kept in −20 °C. Three weeks later, another boost (300 μg of antigen) was injected subcutaneously to the same rabbit. Fifteen days later, blood was collected from the marginal ear vein. Centrifugation was performed as above for serum isolation. In scheme 2, two weeks interval between immunizations was applied instead of three weeks interval used in scheme 1.

The anti-nodavirus IgG was purified using the Nab^TM^ spin columns, following the manufacturer’s instructions. Briefly, the samples were diluted 1:1 in binding buffer (0.1 M Phosphate buffer, 0.15 M NaCl). The columns were centrifuged (1000 × g, 1 min, room temperature (RT)), the flow through was discarded and were equilibrated with 2 mL binding buffer, followed by centrifugation (1000 × g, 1 min, RT). The procedure was repeated once. Subsequently, the bottom ends of the columns were closed, the samples were added and the columns were stirred end-over-end (10 min, RT). After centrifugation, the flow through containing non-bound serum components was collected and the columns were washed 3 times with 2 mL binding buffer. One mL of elution buffer (Glycine 0.1 M, pH = 2–3, ×3 times) was added to the columns, the fractions were collected in tubes containing 100 μL of neutralization buffer (Tris 1 M, pH = 8–9) and stored in −20 °C. Finally, the columns were regenerated with 3 mL of elution buffer (×2 times) and stored with 3 mL of storage buffer (0.02% NaN_3_ in PBS). To avoid possible denaturation of purified antibodies due to sensitivity to elution buffer, purified antibodies were desalted using Zeba^TM^ Spin desalting columns. Briefly, the columns were centrifuged at 1000xg for 2 min, RT. Ultrapure water was added in each column and centrifugation was repeated twice. Samples were placed in each column and columns were centrifuged in order to collect the purified samples. The antibody concentrations were measured using the micro BCA protocol.

A specific ELISA was utilized for anti-nodavirus polyclonal antibodies determination in rabbit serum. ELISA plates (96-well flat bottom) were coated with 100 μL of 0,001% poly-L-lysine in bicarbonate/ carbonate buffer, pH 9.6, (incubation 1 h, RT). The plate was washed 3 times with LSWB and 100 μL of nodavirus suspension were added, followed by incubation for 2 h (RT). Subsequently, 50 μL of 20 mM glutaraldehyde in PBS were added in each well and the incubation was continued for 20 min (RT). The plate was washed with LSWB (3 times) and then blocked with 200 μL 10% BSA in LSWB, for 18 h, at 4 °C. Following plate washing (5 times with HSWB), 100 μL of serially diluted rabbit anti-nodavirus serum (1:10, 1:100, 1:1000 and 1:10000 in PBS/ 10 mL/L Tween-20), were added (incubation 1 h, RT). The plate was washed with HSWB and 100 μL of anti-rabbit IgG-HRP antibody was added; diluted in blocking buffer (1/100, 1/500, 1/1000 and 1/5000), and incubated for 1 h at RT. The plate was washed with HSWB and 100 μL of chromogen (TMB) was added for 3 min. The reaction was stopped using 50 μL of 0.5 M H_2_SO_4_. The optical density was measured at 450 nm in spectrophotometer.

### Preparation of anti-nodavirus antibody conjugated Au NPs

An aliquot of 30 nm Au NPs solution (i.e. 1 mL, 1.8 × 10^11^ particles/mL) was adjusted to pH 9 by adding the appropriate amount of 200 mM borax solution. In parallel, 4 µg of polyclonal anti-nodavirus antibody (2.3 µg/µL) were diluted in 200 µL of 20 mM borax solution and added to the nanoparticles solution, gradually by stirring (4 times × 50 µL). The mixture was incubated for 45 min at RT, and 100 µL of 10% BSA in 20 mM borax solution were added. The final solution was incubated for 10 min at RT, and the excess of reagents were removed by centrifugation at 4,500 × g for 60 min. The supernatant was discarded and the pellet was re-dispersed in washing solution (1 mL 1% BSA in a 2 mM borax solution), followed by centrifugation at 4,500 × g for 10 min. The supernatant was discarded once again, and the red pellet was re-dispersed in 100 µL of an aqueous solution containing 0.1% BSA and 0.1% NaN_3_ in 2 mM borax. All incubation steps were performed in darkness. Finally, the functionalized antibody Au nanoparticles were stored at 4 °C^26^.

### Flocculation studies

Gold colloid suspension (0.3 pmol/ 1 mL) adjusted to pH 8.5, was pipetted into a series of 1.5 mL siliconized tubes. Colloidal gold and antibody (5–60 ng/µL) solutions were mixed and the Au functionalization protocol was applied as described above. Determination of the optimal antibody amount for Au nanoparticles conjugation, was based on light absorption measurements. For that reason each tube received 10% NaCl and was shaken for 5 min. Absorption of each tube at 527 and 580 nm was determined 10 min later.

### Preparation of the nodavirus lateral flow biosensors

The dry reagent lateral flow biosensors (4×60 mm) consisted of a cellulose immersion pad, a glass-fiber conjugate pad, a nitrocellulose diagnostic membrane, and a cellulose absorbent pad. The parts were assembled on a plastic adhesive backing as follows: the diagnostic membrane was placed on center of a laminated card by the manufacturer; the conjugate pad was placed below the membrane, the immersion pad was placed below the conjugate pad, and the absorbent pad was positioned above the membrane. Each part overlapped 2 mm to ensure that the solution could migrate through the biosensor. The TLC applicator was employed to construct the test zone (TZ) and the control zone (CZ) by loading polyclonal anti-nodavirus antibody (anti-pN) and anti-rabbit IgG antibody (anti-R) on the membrane, respectively. The anti-pN and anti-R zones were constructed at distances of 15 and 20 mm from the edge of the membrane, respectively. In details, for the anti-pN zone, a solution consisting of 250 mg/L purified polyclonal anti-nodavirus antibody, 50 mL/L methanol, and 20 g/L sucrose in freshly prepared 100 mM NaHCO_3_ buffer (pH 8.5) was loaded at a density of 500 ng per LFB, with dispensing velocity 60 nL/s. For anti-R zone, a solution containing 500 mg/L anti-rabbit IgG antibody, 50 mL/L methanol, and 20 g/L sucrose in 100 mM NaHCO_3_ buffer (pH 8.5) was loaded at a density of 500 ng per 4-mm membrane, with dispensing velocity 250 nL/s. The membrane was dried in an oven for 1 h at 80 °C, and the sensors were assembled as described before. LFBs with a 4-mm width were cut using a Guillotine cutter and stored dry, at ambient temperature^8,26^.

### Lateral flow biosensor detection assay of nodavirus virions

For detection of nodavirus virions, brain tissue samples were homogenized and centrifuged as described in section “Virus preparation and titration”. Alternatively, aliquots of SSN-1 cell culture supernatant infected with nodavirus were used. The samples were directly applied to the conjugation pad next to 7.5 µL of polyclonal anti-nodavirus conjugated Au NPs. The immersion pad of the biosensor was then dipped into 250 μL of the developing solution. The visual detection was completed within 30 min. Longer times did not affect the assay results^8^. After completion of the assay, the LFBs were scanned with a desktop scanner and the band densities were quantified with ImageJ software^30^.

## Results and Discussion

### Assay principle

**Figure 1:**
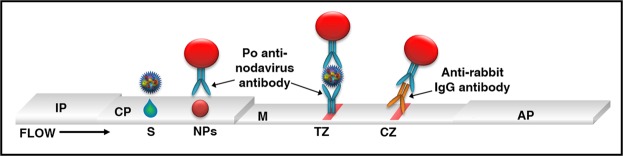
Principle of the nanoparticle-based lateral flow biosensor for detection of nodavirus virions (LFB). Side view of the lateral flow biosensor. IP: immersion pad; CP: conjugation pad; M: diagnostic membrane; AP: absorbent pad. A test zone (polyclonal anti-nodavirus antibody (TZ) and a control zone (anti-rabbit IgG antibody (CZ)) have been immobilized on the biosensors’ diagnostic membrane. The sample contains the nodavirus particles (virions) and is applied on the conjugation pad next to functionalized gold nanoparticles (Au) with polyclonal anti-nodavirus antibody. The biosensor is dipped in the developing buffer, the sample and the nanoparticles are captured on the test zone and the positive signal is visualized, as a red zone. The excess nanoparticles bind to the control zone of the biosensor. The assay components are not in scale.

The principle of viral particles detection based on lateral flow biosensor with functionalized nanoparticles is presented in Fig. 1. Nodavirus intact particles (virions), obtained either from SSN-1 cell culture supernatants or homogenized tissue samples, are directly applied on the LFB. The biosensor is dipped in a developing solution, which is transferred through the LFB by capillary action. The solution rehydrates the anti-nodavirus conjugated gold nanoparticles, and the virion – anti-nodavirus conjugated nanoparticles complex is captured from immobilized anti-nodavirus antibody at the LFB test zone. The resulted accumulation of Au NPs generates a characteristic red line. Proper function of the biosensor is confirmed by immobilization of excessive Au NPs in captured anti-rabbit IgG (control zone) forming a distinct red line. Absence of virus particles in the sample results in the formation of the control zone only.

The proposed LFB is based on the well-established immune reaction between antibody functionalized gold-nanoparticles and virions, which serve as antigens. Since each virion surface i.e. the virus coat protein, contains multiple antigenic sites (e.g. B-cell epitopes)^31^, a sandwich format was implemented for virion detection assay. It has been suggested that a sandwich ELISA resulted in optimum quantitation results, with higher reproducibility and greater detectability range when it was compared with an antigen-immobilized ELISA for nodavirus particles detection^32^, therefore the sandwich format was chosen in the present project. Moreover, the sandwich format has been successfully implemented in various other formats for low cost portable devices, e.g. nanoparticle-mediated photothermal assays with common thermometer as the signal reader^33^, indicating its appropriateness for methodologies with on-site applications. Therefore, polyclonal antibodies to NNV were immobilized on the test zone of the biosensor as well as on the gold nanoparticles.

### Production and reactivity of rabbit anti-nodavirus polyclonal antibodies

The anti-rabbit IgG concentration for the ELISA assay was tested in the range of 1:100, 1:500, 1:1000 and 1:5000 dilution. The serum dilution range was 1:10, 1:100, 1:1000 and 1:10000. The results are presented in Fig. S1a. To optimize the polyclonal antibodies efficiency, the rabbit serum was purified with the appropriate columns and specificity of the purified and desalted antibodies was tested with ELISA. Optical densities (OD_450_) for a representative fraction was 1.914 (elute 1) and 2.038 (elute 3) versus 1.752 of the unpurified serum. The use of 1:1000 dilution of the anti-rabbit IgG antibodies was selected since it results in similar signal intensity with higher amount of the antibody, for the same serum dilution. The immunization scheme 1 consisted of two parts: 3 times of antigen administration with 3 weeks intervals between immunizations and a 4^th^ immunization followed 3 weeks later. Antibody levels were quantified with a specific ELISA, and the final immunization was performed to raise higher anti-nodavirus antibodies titer (OD_450_ = 0.885 for 3 immunizations vs OD_450_ = 1.471, for 4 immunizations). In parallel, another female rabbit was immunized with scheme 2 which allowed a 2 weeks interval between the immunizations. As shown in Fig. S1b, scheme 2 raised even higher titer of IgG anti-nodavirus antibodies compared to scheme 1. Therefore it was used for antibody production.

### Optimization of the Au nanoparticles anti-nodavirus conjugates

Colloidal gold nanoparticles were selected since they are the most common used nanosized labels for lateral flow biosensors, occupying more than 90% of the respective market in immunochromatographic assays, since they can be biolabeled with simple procedures, have excellent optoelectronic properties, have very low toxicity and are easily synthesized with low cost^34^. Conjugation of the anti-nodavirus antibody on the Au NPs was optimized in terms of: usage of a monoclonal versus the polyclonal anti-nodavirus antibody, the size of the used particles, the anti-nodavirus antibody amount on the nanoparticle conjugates and the amount of the conjugates that was applied on the lateral flow biosensors.

#### Effect of the use of polyclonal instead of a monoclonal antibody for Au NPs functionalization

**Figure 2:**
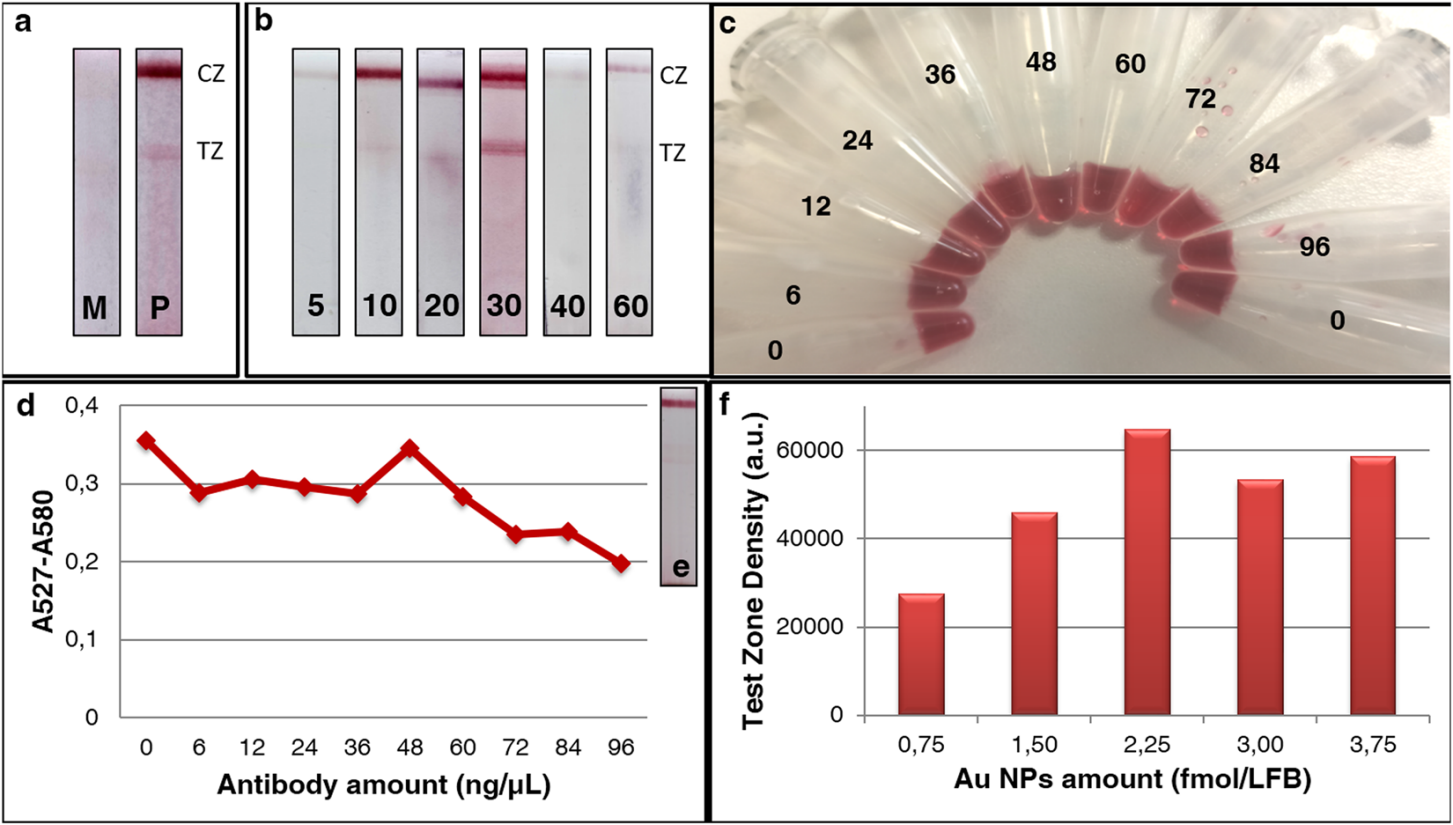
(**a)** Effect of the use of monoclonal (M) versus polyclonal (P) antibody for Au NPs functionalization. (**b)** Effect of the Au NPs size on anti-nodavirus conjugation reaction. The used sizes were 5, 10, 20, 30, 40 and 60 nm. (**c**) Gold NPs conjugates with different antibody concentrations (ng/µL). (**d**) Light absorption of conjugates at different antibody concentrations. (**e)** Representative lateral flow biosensor of anti-rabbit IgG control zone with polyclonal anti-nodavirus conjugated gold nanoparticles. (**f)** Effect of the anti-nodavirus Au nanoparticles conjugate amount on LFB. CZ: control zone, TZ: test zone.

The conjugation of a monoclonal anti-nodavirus antibody on the nanoparticles was tested in order to increase specificity. As shown in Fig. 2a, the monoclonal antibody failed to provide functionalized Au NPs since not even the CZ was visible. On the contrary, implication of a polyclonal anti-nodavirus antibody in the conjugation reaction resulted in a discrete signal in the test zone of the LFB and a strong signal in CZ.

#### Effect of the gold nanoparticles size

The Au NPs size has important effects on the particles optical properties, the affinity of the conjugates with the used antibodies, their valency and the antigen binding kinetics^24^. Large sized particles develop steric hindrance in a high degree, affecting interactions of the target with a labelled antibody, which could impede the fluidity in the strip. On the other hand, small nanoparticles, are not optimal for visual signals formation^35^. To determine the size where the signal would be optimal, commercially available nanoparticles were studied. Gold nanoparticles stabilized either in citrate (40 nm) or in PBS buffer (5, 10, 20, 30, 60 nm) were conjugated with the polyclonal anti-nodavirus antibody. Formation and intensity of TZ and CZ on the LFB was assessed for evaluation of the particles optimum size for the nodavirus LFB. Figure 2b proves that all PBS stabilized particles were successfully conjugated with antibodies while no conjugation occurred on the citrate 40 nm nanoparticles. Therefore, Au NPs stabilized with PBS buffer facilitates proteins (antibodies) conjugation while the citrate buffer is more efficient on conjugation of SH-tailed oligonucleotides^8^. Use of 5 and 60 nm NPs resulted in faint control zones. The 10 and 20 nm formulations formed strong control zones but the test zones were faint possibly due to difficulty in interaction with the larger nodavirus particle. The optimum signal was achieved with 30 nm Au NPs, a size which is similar to the virus size. That size seems to be optimum for conjugation with different antibodies for different analytes intended for use on LFBs, e.g. anti-*E.coli* monoclonal antibody^35^ or anti-biotin antibody^26^, indicating that nanoparticles in the range of 30–40 nm are better for antibody conjugation than smaller or bigger particles.

#### Effect of anti-nodavirus antibody amount on Au NPs conjugates

The antibody amount used for nanoparticles functionalization is a crucial factor for optimal LFB performance. Variation in the antibody amount in the surface of the nanoparticles will affect its density on the conjugate, affecting the binding degree between the antigen and the conjugate. Thus, the assays’ detection limit can be changed based on the various binding efficiency. The minimum antibody concentration which inhibits nanoparticles aggregation in the presence of excess salt (10% NaCl), is usually chosen as the optimum amount for Au NPs conjugation^24^. Several antibody amounts were studied and the optimal antibody concentration for the particle functionalization was determined by comparing the absorption between 527 and 580 nm (A_527_–A_580_), in the presence of 10% NaCl. The resulted conjugates are presented in Fig. 2c, where all nanoparticles appear to be of the same color.

The gold nanoparticles stability in the presence of concentrated NaCl is indicated by the flocculation curve, which is an indirect measure of the immobilized protein surface coverage degree. A hydrated charged corona of proteins on the nanoparticles is formed by adsorption on their surface, which inhibits the particles aggregation, mostly due to steric hindrance and electrostatic repulsive forces. Addition of a concentrated NaCl solution inverts the Au NPs protection by uncovering their surface, leading to aggregation^11^. The resulted flocculation curves indicated that 40 ng/µL was the optimal antibody amount, which was therefore used throughout the project (Fig. 2d). Application of that conjugate on the LFB resulted in a strong signal in the control zone, confirming the successful conjugation of anti-nodavirus antibody to Au nanoparticles (Fig. 2e).

#### Effect of anti-nodavirus Au NPs conjugate amount on LFB

The Au NPs amount on the conjugate pad was evaluated, in order to achieve satisfactory CZ and TZ intensity of the biosensor, combined with minimum background membrane coloration^24^. The nanoparticles amount was optimized in the range of 0.75–3.75 fmol per LFB. The use of 2.25 fmol of Au nanoparticles per LFB resulted in the optimum signal (Fig. 2f). Less conjugate amount resulted in lower band intensities possibly because low conjugate content is inadequate for binding of specific epitopes amount on highly concentrated antigens. Use of higher amounts of particles had a slight signal decrease, as expected since higher conjugate cause epitopes blocking on the antigen surface, and inhibits their binding in the LFB test and control zones^11^. Based on these observations, 2.25 fmol (7.5 μL of the prepared solution) of gold nanoparticles conjugates, corresponding to 7.5 μL of the prepared solution, were used in all subsequent experiments.

### Optimization of LFB test zone construction

Construction of the lateral flow biosensor is extremely important since a large number of parameters would affect the sensitivity and specificity of the assay. Several factors were studied including the developing solution composition, the type of the used diagnostic membrane, the dispensing velocity, the use of a polyclonal instead of monoclonal antibody and the immobilized antibody amount for TZ construction.

#### Effect of the composition of the developing solution

The developing solution composition has great impact on the antibody-antigen interaction on the biosensor. Ideally, the developing solution facilitates the nanoparticles and sample mixture movement along the biosensor with flow rates that allows test completion in short time providing, in parallel, sufficient time for virion interaction with the modified NPs. Moreover, the developing solution should also prevent the non-specific nanoparticles absorption by the LFB membrane^36^. Glycerol increases the mixture viscosity causing a slower flow rate. Addition of several surfactants (SDS, Tween-20, SDS, etc) or proteins (e.g. BSA) enables the particles movement from the conjugate pad. Eleven different formulations were studied and their detailed composition is described in Table 1, while the respective results are presented in Fig. S2. The use of high pH buffer or BSA did not result in any specific signal formation (Fig. S2a). Two formulations were optimal for non-specific signal elimination: i) 8G/1S/P, and ii) 6G/1S/0.1T/P. The formulation that contained 80% glycerol resulted in prolonged running times (∼60 min); therefore it was excluded from further studies. It was observed that Tween-20 concentration seemed decisive for the biosensor specificity. For that reason, increasing Tween-20 concentrations were tested in the range of 0.1–1% and the optimum results were obtained with 0.2% Tween-20 in developing solution (Fig. S2b). Concluding, the optimum developing solution for background elimination, rapid assay times, and specific bands was obtained using 6% glycerol, 0.2% Tween-20 and 1% SDS in 1×PBS, pH 7.4.

**Table 1:** Composition of developing solutions tested with Nodavirus LFB. S: SDS, P: PBS, G: glycerol, T: Tween-20, N: NaHCO_3_, B: BSA.

Developing solution	SDS (%)	Glycerol (%)	Tween-20 (%)	PBS pH 7.4	NaHCO_3_ pH 8.5	BSA (%)
1 S/P	1	−	−	+	−	−
2 G/1 S/P	1	2	−	+	−	−
4 G/1 S/P	1	4	−	+	−	−
6 G/1 S/P	1	6	−	+	−	−
8 G/1 S/P	1	8	−	+	−	−
6 G/1 S/1 T/P	1	6	1	+	−	−
6 G/1 S/0.1 T/P	1	6	0.1	+	−	−
6 G/0.1 S/0.1 T/P	0.1	6	0.1	+	−	−
6 G/1 S/0.1 T/1B/P	1	6	0.1	+	−	1
6 G/1 S/N	1	6	−	−	+	−
6 G/1 S/1 T/N	1	6	1	−	+	−

#### Effect of the antibody dispensing velocity

**Figure 3:**
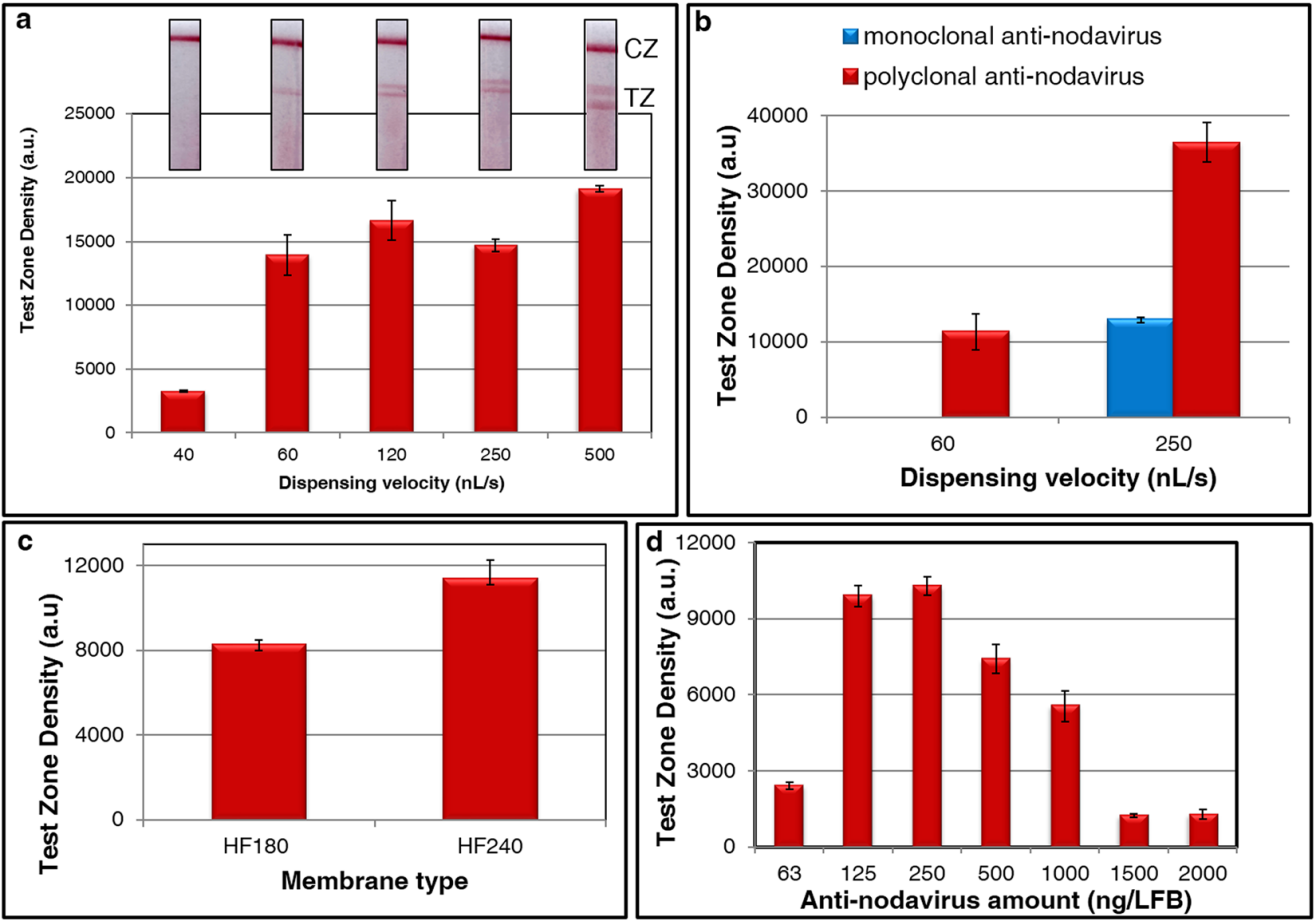
(**a)** Effect of the dispensing velocity on the LFB test zone signal intensity. (**b)** Comparison of the use of monoclonal anti-nodavirus antibody versus a polyclonal anti-nodavirus antibody, in different dispensing velocities (60 and 250 nL/s) for construction of the LFB test zone. (**c)** Effect of the nitrocellulose membrane type on the LFB test zone signal intensity. (**d**) Effect of the polyclonal anti-nodavirus antibody amount used for construction of the LFB test zone. CZ: control zone, TZ: test zone.

The dispensing velocity for construction of the TZ based on the polyclonal anti-nodavirus antibody was subsequently tested (Fig. 3a). The antibody dispensing velocity is an essential parameter for production of a uniform immobilized test zone. In general, high volatile solutions (e.g. acetone) are dispensed with high velocity (>200nL/s) whereas low volatile solutions (water, ethanol) are sprayed with low velocity (<120nL/s). Based on previous studies for antibody immobilization on nitrocellulose membranes^37^, a methanol containing solution (50 mL/L) was used. Methanol results in medium volatile solutions and the use of 250nL/s dispensing velocity was optimum for the uniform formation of the anti-rabbit IgG control zone. However, the same dispensing velocity resulted in the formation of two ‘pseudozones’ when the anti-nodavirus antibody solution was sprayed on the membrane. It seems that high dispensing velocity creates a gap in the middle of the spraying zone, which doesn’t allow proper absorption of the antibody solution from the membrane, perhaps due to non-uniform evaporation. The use of lower dispensing velocity resulted in a single uniform test zone and the velocity of 60nL/s was chosen since it resulted in the optimum signal.

#### Effect of the use of polyclonal instead of monoclonal anti-nodavirus antibody

In order to enhance the LFBs’ specificity, a monoclonal anti-nodavirus antibody which was commercially available was used instead of the produced polyclonal antibody. It is obvious in Fig. 3b, that the monoclonal antibody did not produce visual signal whereas use of the polyclonal antibodies led to high signal formation, since more antigenic epitopes interacted with higher antigen amounts and were subsequently immobilized.

#### Effect of the type of the diagnostic membrane

The porosity of nitrocellulose membranes plays a critical role for the specificity and the sensitivity of a lateral flow biosensor because it influences the capillary flow rate. Two membranes, the HF180 and HF240 (Millipore) were tested for the nodavirus LFB development, with corresponding migration times of 3 and 4 min, respectively. As shown in Fig. 3c, the signals of the LFBs which were based on the HF240 membrane were more intense than the signals obtained with the HF180 membrane. Thus, longer migration time is favorable for the sandwich-type complexes on the LFB, in accordance with independent observations^38^.

#### Effect of the immobilized antibody amount

The anti-nodavirus antibody amount immobilized on the biosensor test zone was examined. 250 ng of antibody per 4-mm LFB were used to obtain the optimum signal (Fig. 3d). The use of 125 ng/µL for antibody immobilization resulted in slightly decreased signal, which was significantly lower when 62.5 ng/µL of the antibody were applied. Interestingly, the signal decreased as the antibody concentration increased. The use of high amounts of antibodies on lateral flow biosensors could cause the so-called “hook effect”. The prozone or hook effect is basically a false negative result of a particular analyte, when an antibody has very high concentrations. If too many antibodies that can bind to the antigen are present, then the antigenic sites are coated by these antibodies, and few or no conjugated Au nanoparticles directed toward the analyte are able to bind more than one antigenic particle. Since the antibodies do not bind to antigens, no signal is formed. Therefore, the result is a false negative^39^.

### Analytical parameters of nodavirus biosensor

#### Detectability with the nodavirus biosensor

**Figure 4:**
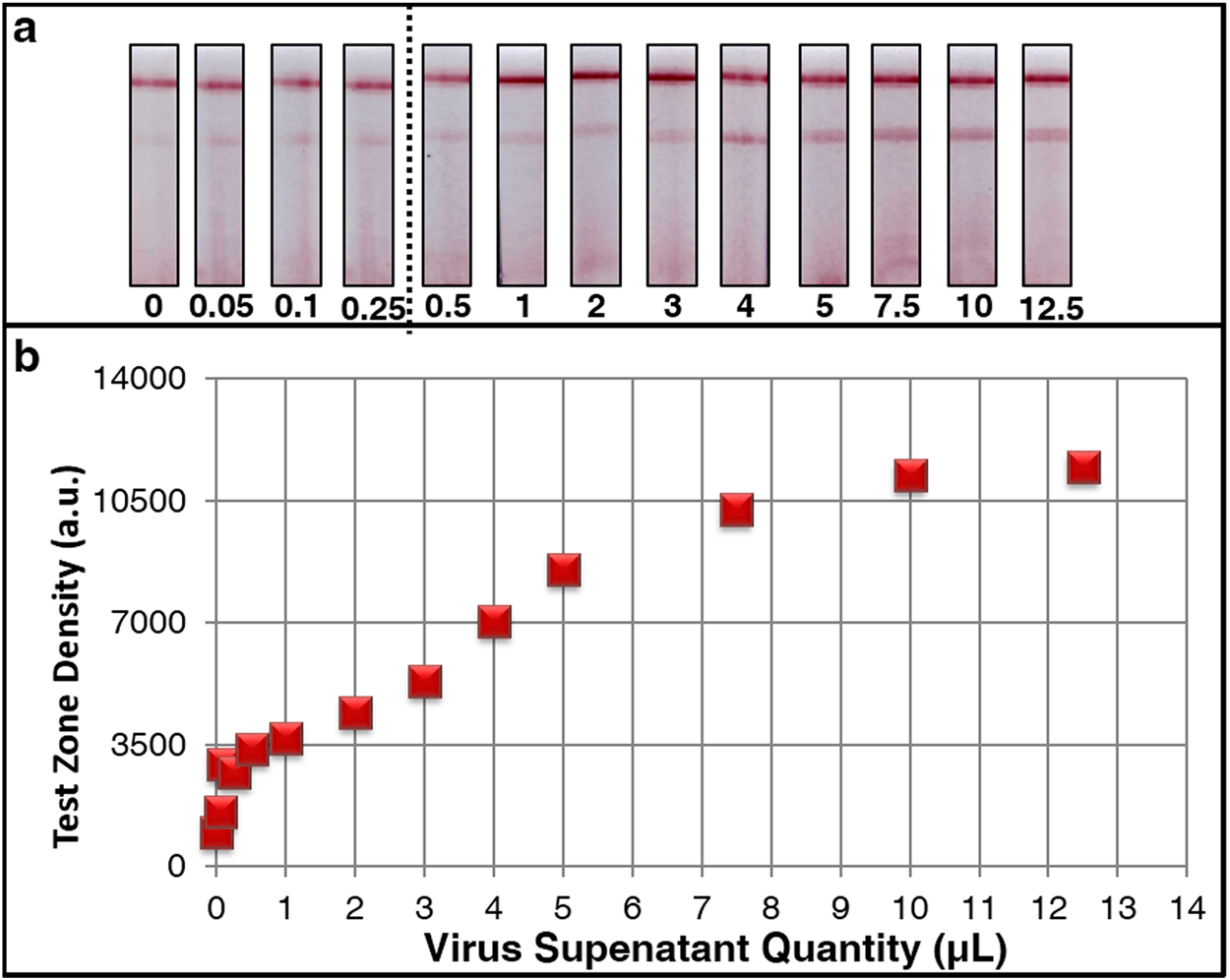
(**a**) Typical images of the LFBs after applying different amounts of nodavirus containing cell culture supernatant. The dashed line indicates the limit of detection. (**b**) Graph of the intensities of the LFBs test zones versus the nodavirus containing supernatant amount.

Nodavirus LFB detectability was assessed by samples consisting of decreasing concentrations of nodavirus infected SSN-1 cell culture supernatants. All samples were studied by applying the optimized experimental conditions. In details, supernatant volumes ranging from 0.05 to 12.5 µL of supernatants were applied on LFBs. The respective LFBs are shown in Fig. 4a,b. The lowest detectable amount of nodavirus containing supernatant was 0.5 µL. To define a positive result and discriminate from any background signal, the signal/noise (S/N) ratio 3 (S/N = 3) was used. The signal definition refers to the TZ optical intensity of each detected sample, whereas noise is the produced TZ intensity when using culture medium alone. The lowest amount corresponded to 0.006 TCID_50_ (supernatant titer was 11.3 TCID_50_/mL) which is the measure of infectious virus titer. High amounts of virus supernatant seem to result in stable densities.

#### Nodavirus LFB reproducibility

Reproducibility is one of the most crucial parameters of the LFB evaluation procedure. For that reason, it was assessed with a constant amount of nodavirus containing SSN-1 cell culture supernatant (5 µL). Five biosensors were tested with the nodavirus supernatant for studying test and control zones. All LFBs were prepared in variable batches. The LFBs are shown in Fig. S3, and analysis by densitometry resulted in coefficient of variation (CV) of 5.7% for the test zones and 6.2% CV for the control zones.

#### Nodavirus LFB specificity

**Figure 5:**
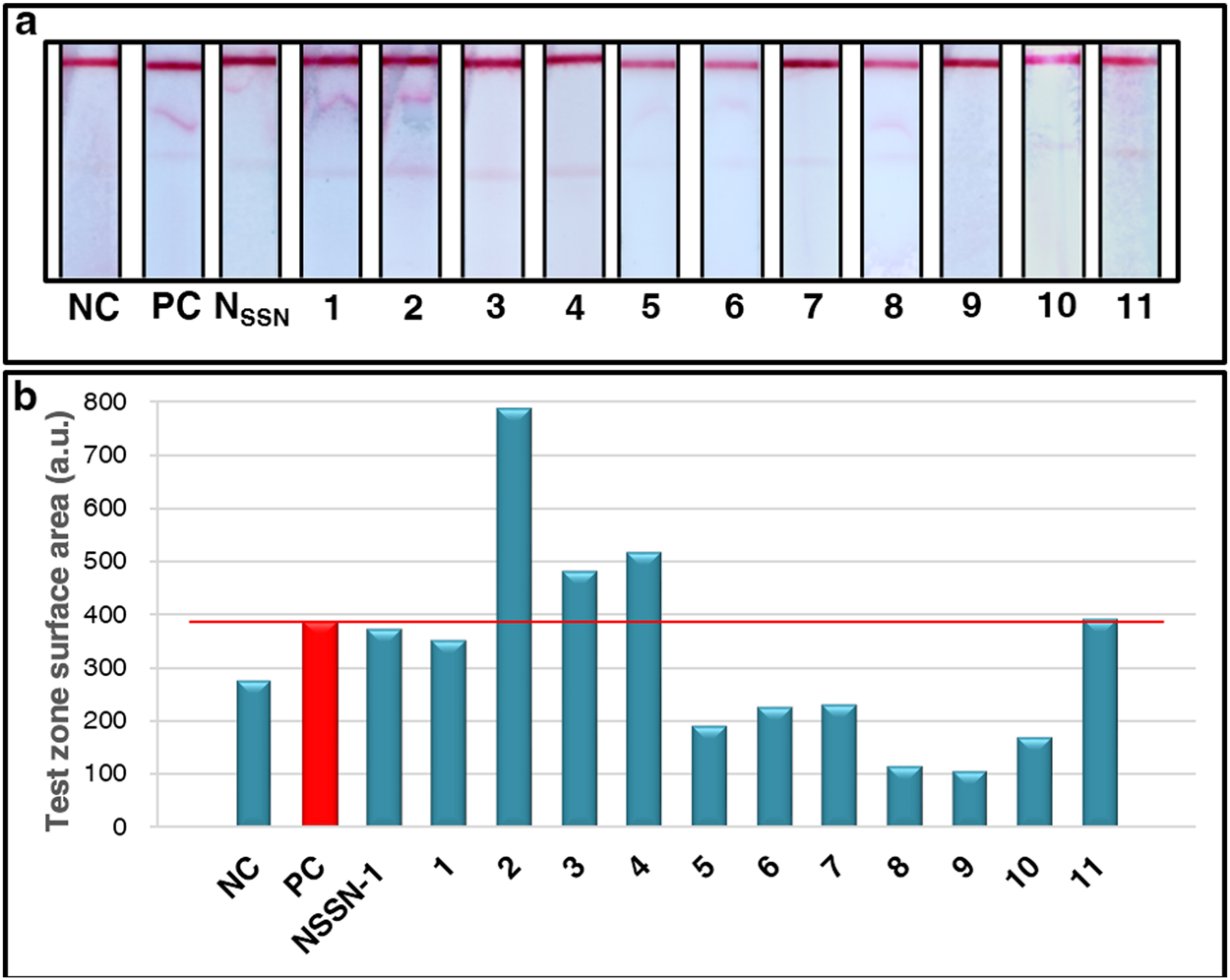
(**a**) Visual detection of Nodavirus particles (virions) with lateral flow biosensors. NC: Negative control (developing solution); PC: Positive control (0.5 µL of nodavirus containing cell culture supernatant); N_SSN_: SSN-1 negative control (non-infected SSN-1 cell culture); N1, 5–10: Negative samples; S2–4, 11: Nodavirus positive samples. (**b**) Graph of the intensities of the LFBs test zones for each tested sample.

Specificity of nodavirus biosensor was assessed by studying the assay cross-reactivity with non-infected SSN-1 cell culture supernatant and homogenized tissues of non-infected fish (Fig. 5). Only the samples which were characterized positive by cell culture resulted in detectable signal. Developing solution-only negative controls were also tested in each run.

Concluding, the analytical performance of the proposed assay can be summarized as follows: 6 × 10^−3^ TCID_50_ of cell culture supernatant was detectable, by using a standard centrifugation step and the proposed biosensor. The assay is specific for infected fish and highly reproducible. In Table 2, the assay sensitivity of previously reported methods for nodavirus virion detection by sandwich/ capture ELISA, as well as nodavirus detection with lateral flow assays in general, are summarized. Even though comparison of these methods’ sensitivity cannot be highly accurate, since the limit of detection is determined by slightly different way in each case, the present assay seems to have higher sensitivity for virion detection in comparison to ELISA methodologies and the mAb/LFIA^40^. Detection limits of lateral flow assays based on nucleic acid amplification are also included in the table, for method estimation purposes.

**Table 2:** Comparison detection limits of fish nodavirus detection methodologies, i.e. ELISA and lateral flow assays.

	Sensitivity		Reference
	Cultured virus	Infected tissue	
**Capture/sandwich ELISA**	6.5 × 10^4^ TCID_50_		^42^
	2–3 × 10^3^ TCID_50_		^43^
	35 × 10^5^ TCID50/ mL		^44^
**mAb/LFIA**	10^5.05^ TCID_50_/100 μL		^40^
**Virion LFB**	**6 × 10**^**–3**^ **TCID**_**50**_ **/ 0.5 μL**		**Present study**
**RT-PCR/ LFB**		135 pg total RNA	^8^
**RT-LAMP–LFD**		2 pg total RNA	^45^
**CPA-LFD**		10^1^ copies/μL	^46^

### Detection of nodavirus infection on *D. labrax* samples with the biosensor-based assay

The proposed LFB enabled nodavirus detection in *D. Labrax* samples with minimum pre-processing (i.e. manual brain homogenization and separation of the insoluble fraction by centrifugation or SSN-1 cell culture supernatant). The samples supernatants were applied directly to the nodavirus biosensors, which were run and scanned. Eleven infected (positive; n = 4) and non-infected (negative; n = 7) *D. labrax* samples were applied on the LFB (Fig. 5). All results were visualized within 30 minutes. All tested samples were verified with SSN-1 cell culturing. Five samples were originated from Saronikos Gulf area (S1, 2, 6, 8, 9), 2 samples were from Korinthian Gulf region (S3–4), 1 sample was collected from Amvrakikos Gulf (S7) and 3 samples were originated from aquacultures in Cyprus (S5, 10, 11). A negative control, containing only developing solution, and a positive control, containing 0.5 µL of nodavirus containing cell culture supernatant (corresponding to the limit of detection), were included in each run. The use of the cell culture supernatant in an amount corresponding to the assays detection limit was intended to be used as a visual “yardstick”, a comparison measure to indicate which signal could be positive. If the positive control was intended to be used just to confirm the LFBs’ proper operation more supernatant could be applied to provide more intense signal. We observe that in the LFBs with positive signal the gold nanoparticle conjugates appear to bind non-specifically between the test and the control zones. In any case that non-specific binding is not crucial for the nodavirus presence assessment, since the test zone is clearly positive or negative. Most of the non-infected samples (S5–10) did not show any signal in the TZ. The infected samples resulted in positive signals with varying intensities above the positive control signal, which correlates with different amounts of nodavirus in the sample. One negative and one positive sample (S1 and S11, as characterized by SSN-1 cell culturing) resulted in similar faint zones. In order to decide the disease state of each sample, the test zones were quantified with the publicly available, easy to use ImageJ software, based on scanned LFB images. Quantification confirmed that S1 was negative and S11 positive with very low virus load (Fig. 5b). In the case of such ambiguous results on the field, the LFBs images could be photographed and quantified with ImageJ with a smartphone. The scanned images of the LFBs were independently evaluated by a second person, and were compared to LFB images with known outcomes (positive and negative).

## Conclusions

Cost-effective and labor-saving methods for fish infectious diseases identification have attracted an increasing interest to aquaculture industry in recent years, due to their high economic impact. The development of a highly promised robust and rapid lateral flow biosensor has been reported in the present study. The proposed LFB can be used for simple and accurate screening of nodavirus detection in various fish species. To enable on site analysis of nodavirus, we developed and optimized a paper LFB based on gold nanoparticles for easy, specific and sensitive visual detection of nodavirus viral particles (virions) in biological samples. The target virions can be simply detected by the red colored signal formation in the LFBs test zone. The LFB permits the visual detection of nodavirus particles within 30 minutes without using any instrumentation. The total assay starting including tissue homogenization, centrifugation and virion detection with the biosensor was accomplished in less than 60 min. The total cost of the assay is less than 3 €. Undergoing studies by our research group include complete validation of the nodavirus biosensor on fish culture facilities, and efforts to improve the biosensors’ sensitivity of the LFB utilizing dual nanoparticles labels^26,41^ and signal enhancement systems in general. Moreover, the elimination of centrifugation from the sample preparation step is among our future goals to further simplify nodavirus detection on-site. Concluding, the proposed biosensor could become a reliable tool for the rapid screening of questionable samples in aquaculture facilities. As a screening tool it can be used to decide whether a sample is positive or negative, and indicate which samples have ambiguous state of the disease and need further analysis by other methodologies in a laboratory setup. Such a procedure could lower the cost and the analysis time to maintain healthy fishes in aquaculture facilities.

## Supplementary information

Supplementary Information.
