# Supplementary material for: Development of a Nanoparticle-based Lateral Flow Strip Biosensor for Visual Detection of Whole Nervous Necrosis Virus Particles
Source: Sci Rep. 2020 Apr 16;10:6529. doi: 10.1038/s41598-020-63553-z (PMC7162894; doi:10.1038/s41598-020-63553-z)
Supplement: Supplementary file 1 — Supplementary Information. [file 41598_2020_63553_MOESM1_ESM.pdf]

Original Research Paper

Development of a Nanoparticle-based Lateral Flow Strip Biosensor for Visual Detection of Whole Nervous Necrosis Virus Particles

Dimitra K. Toubanaki [a,*], Maritsa Margaroni[a], Athanasios Prapas[b] and Evdokia Karagouni [a]

[a] Immunology of Infectious Diseases Laboratory, Department of Microbiology, Hellenic Pasteur Institute, 127 Vas. Sofias Ave., 11521, Athens, Greece.

[b] Department of Pathology of Aquatic Organisms, Veterinary Center of Athens, 25 Neapoleos Str, 15341, Agia Paraskevi, Greece.

***** Author to whom correspondence should be addressed: E-Mails: dtouban@pasteur.gr; dtouban@gmail.com (D.K.T.)

Tel.: +30-210-647-8825; Fax: +30-210-647-8828

Dimitra Toubanaki: https://orcid.org/0000-0003-2250-3250
Evdokia Karagouni: https://orcid.org/0000-0002-0987-4877

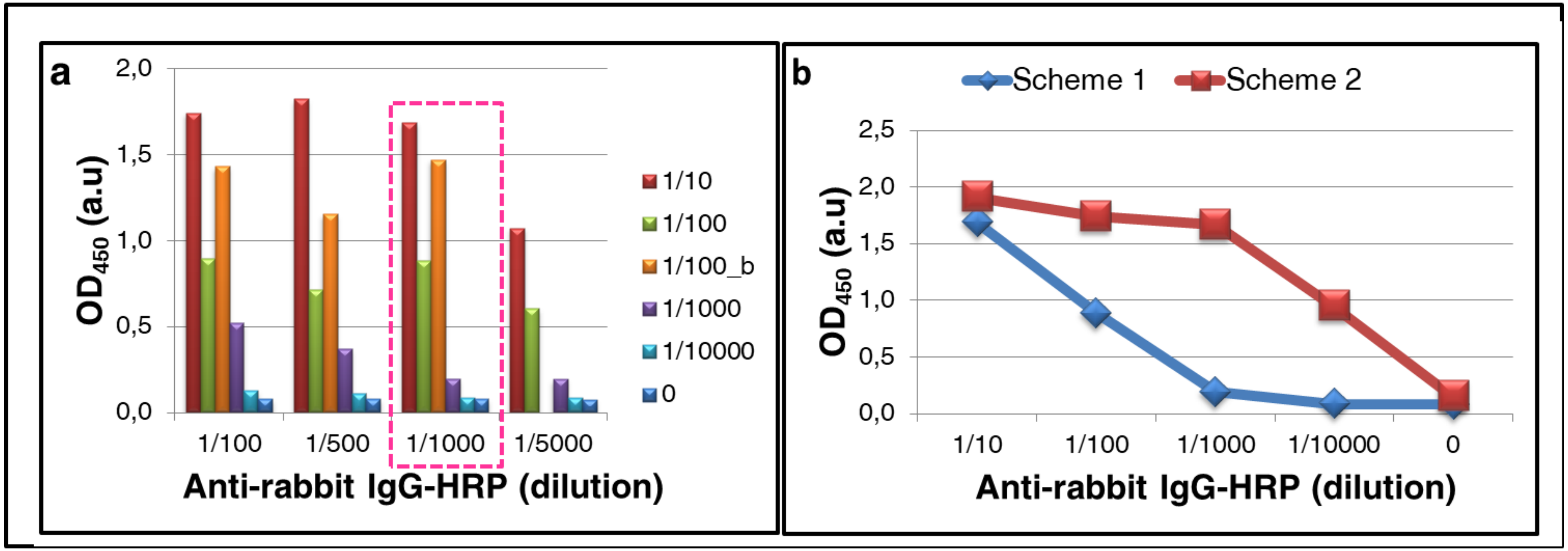

Fig. S1 a. Study of different dilutions (1/100, 1/500, 1/1000 and 1/5000) of anti-rabbit IgG HRP antibody for detection of rabbit IgG antibodies raised in rabbit immunized with nodavirus containing cell culture supernatant (1/10, 1/100, 1/1000, 1/100000 dilution for immunized sera (3 injections), and 1/100 b (4 injections)). **b.** IgG antibodies raised in rabbit immunized with nodavirus after 3 immunizations (Scheme 1: 3 weeks interval, scheme 2: 2 weeks interval). Different dilutions (1/10, 1/100, 1/1000, 1/10000) of rabbit serum antibody were used. Anti-rabbit IgG HRP antibody was diluted 1/1000

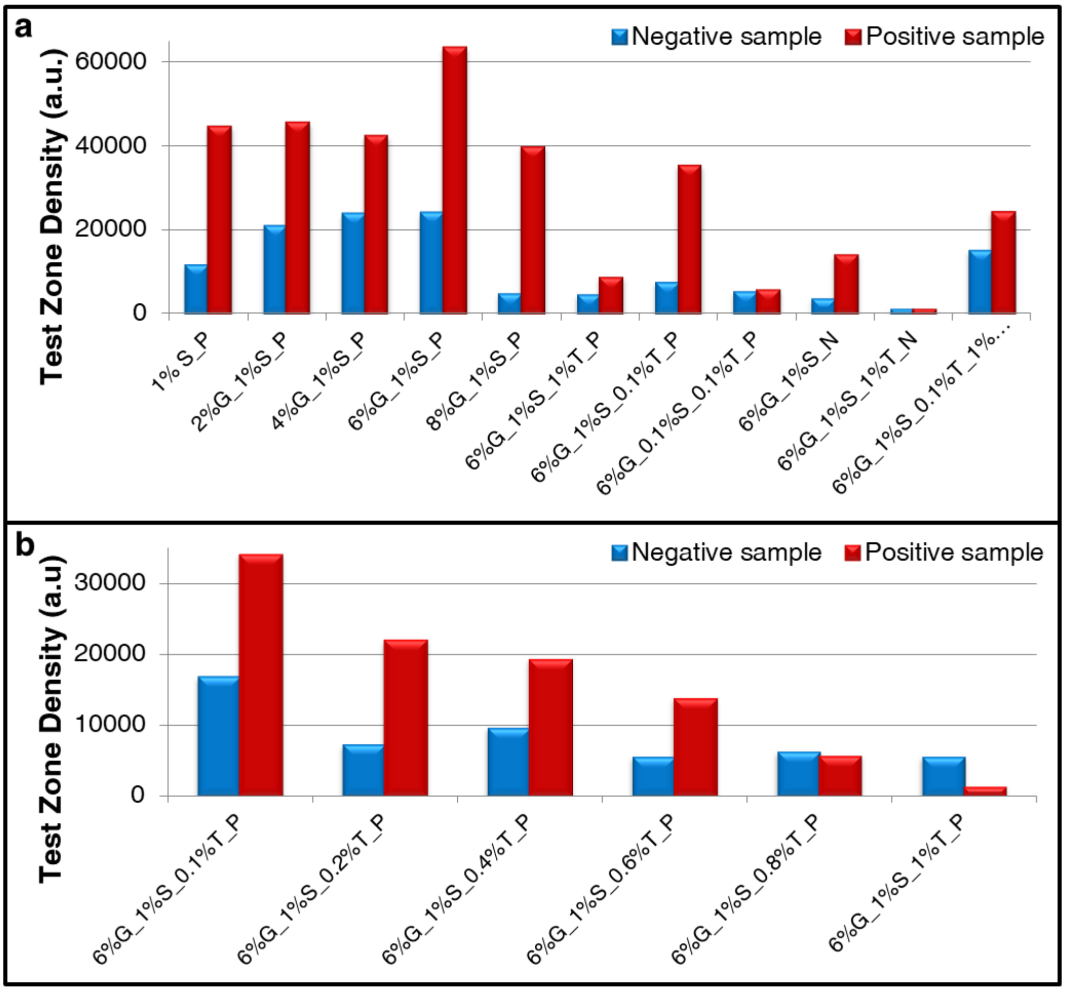

Fig. S2 Effect of the developing solution composition on the LFB test zone signal intensity. S: SDS, P: PBS, pH 7.4, G: glycerol, T: Tween-20, N: $NaHCO_3$, pH 8.5, B: BSA

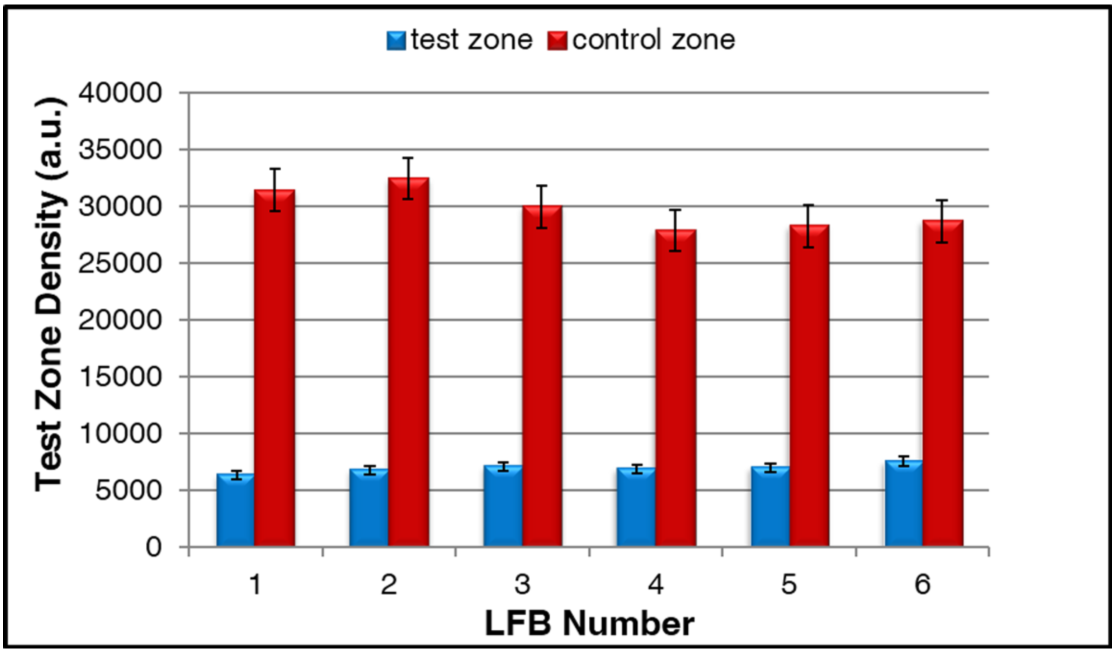

Fig. S3 Reproducibility study of LFBs test and control zones with nodavirus containing supernatant as target (CV_{TZ}: 5.7%, n = 6, CV_{CZ}: 6.2%, n = 6)
